# Climate change and primary health care in Africa – A call for short reports

**DOI:** 10.4102/phcfm.v14i1.3583

**Published:** 2022-05-26

**Authors:** Christian L. Lokotola, Robert Mash

**Affiliations:** 1 Division of Family Medicine and Primary Care, Faculty of Medicine and Health Sciences, Stellenbosch University, Cape Town, South Africa

This editorial introduces a call for short reports on how climate change is impacting primary health care (PHC) in Africa. We are interested in your stories of how climate change is affecting you and how you are adapting or preparing to be more resilient. Share what is happening in your practice and what you have learnt. You may also have contributed to mitigation and more responsible consumption and can also share these stories. We know little about PHC and climate change in Africa, and this is an opportunity to broaden and deepen our understanding.

Human-induced emissions of greenhouse gases have triggered global warming and climate change, which have direct and indirect adverse consequences for human health and well-being.^1^ Consequences include not only frequent heatwaves, rising sea level and ocean temperature, an increase in heavy precipitation and violent storms with flooding but also drought and water scarcity. Climate change contributes to biodiversity loss and extinction of species with disruption of essential ecosystem services, such as food and water quality and quantity. The natural environment that supports health and well-being is being disrupted.^2,3^

The recent report from the Intergovernmental Panel on Climate Change states that global surface temperature is 1.09 °C (0.95 °C to 1.20 °C) higher in 2011–2020 compared with 1850–1900.^4^ There is a greater than 50% likelihood that global warming will reach or exceed 1.5 °C in the near future.^4^ The consequent risks arise from multiple climate hazards occurring concurrently and multiple risks interacting. The extent and magnitude of climate change impacts are larger than estimated in previous assessments.

Climate change is already impacting health in a myriad of ways, leading to increased human and animal mortality and morbidity.^5^ The multiple environmental crises increase the risk of zoonosis, such as the coronavirus disease 2019 (COVID-19) pandemic. Water-borne infectious diseases are more likely with poor water quality and quantity, while vector-borne diseases may increase in some areas as the vectors’ natural habitat changes. For example, malaria may emerge in the highlands with increasing temperatures.^6^

Extreme temperature and frequent heatwaves may affect the body’s thermodynamic system leading to heatstroke and exhaustion.^2^ Air pollution is associated with a higher prevalence of allergens, respiratory diseases and cardiovascular diseases. Extreme weather events, such as cyclones, may be associated with acute trauma, displacement of populations and loss of life.

Food insecurity may be worsened with associated malnutrition as drought impacts farming or climate change impacts the types of crops that can be grown successfully. Other environmental crises such as land conversion and deforestation and loss of soil quality will impact on food production.

Many of the impacts of climate change lead people to consider migration or cause displacement and are associated with an increase in psychosocial stress and mental health disorders.

Although climate change affects all continents, its consequences will not be the same everywhere. Poor people in developing countries are more vulnerable to the impact as individuals and communities. They have limited ability to adapt to the changes or to be resilient in the face of change. This is the situation in many African countries.

In Africa, we are already seeing the impact of climate change.^7^ Notable examples include the impact of multiple cyclones in Mozambique and Malawi. Cyclones have caused loss of life, injury, displaced populations and destroyed infrastructure and livelihoods. In East Africa, prolonged drought has led to food insecurity, migration, water-borne infectious diseases and malnutrition. The recent locust invasion from the Arabian Peninsula has also been linked to climate change. In Nigeria, the conflict has emerged between herders and farmers as climate change alters the growing season for farmers and disrupts what was previously a symbiotic relationship. The 2018 Lancet Commission stated that ‘climate change is the biggest global health threat of the 21st century’, and this threat is already well established on the African continent.^8^

Strong PHC can increase the resilience of communities in the face of climate change.^9^ In Africa, however, PHC is often poorly resourced and underdeveloped. There is an urgent need to develop climate-resilient PHC.

Primary healthcare may need to adapt in multiple ways to the impact of climate change. Primary healthcare must anticipate and prepare for the likely extreme weather events and consequences such as floods or fires, so that it can respond to the emergencies and continue to provide routine services. Strong links to public health, early warning systems, disaster preparedness and emergency medicine will be needed. Infrastructure must be able to withstand these extremes of wind and precipitation. Medication, supplies and equipment should be available even in these extreme situations. Primary health care must also monitor and adapt to changing patterns of disease and morbidity as well as increased pressures from displaced populations and migration.^9^ Migrants are often marginalised and vulnerable to health services and may have different health problems from the local population.

Health systems in the developed world are also a major contributor to greenhouse gases, and the global health system is responsible for 4.4% of all emissions.^10^ Therefore, there is a need for health systems to contribute to the mitigation of climate change by giving attention to the use of energy, water, waste, buildings, procurement, pharmaceuticals, chemicals, transport and food.^11^ Most public sector PHC services in Africa will have a trivial carbon footprint. Nevertheless, they should commit to responsible consumption. There may also be co-benefits to responsible consumption. For example, rural clinics can benefit from solar voltaic power and storage as they are often far from the national grid.

Primary care providers have an important role to play in advocating for change and explaining the impact on human health to policymakers. There is also an imperative to prepare the next generation of health professionals to understand planetary health and the health consequences of exceeding our ecological limits.^12^ We should all be environmental stewards in our personal and professional lives.
